# Residual transmission of HIV infection from mother to child in the Atlantic and littoral departments in Benin

**DOI:** 10.1186/s12887-024-05131-0

**Published:** 2024-10-14

**Authors:** Edwige Hermione Dagba Gbessin, Haziz Sina, René Kpemahouton Keke, Michel Kiréopori Gomgnimbou, Aldric Afangnihoun, Moussa Bachabi, Abdoul-Salam Ouedraogo, Lamine Baba-Moussa

**Affiliations:** 1 NAZI BONI University, Bobo-Dioulasso, 01 BP 1091 Burkina Faso; 2 National Reference Laboratory of Health Program Fighting Against AIDS in Benin (NRL/PSLS), Health Ministry of Benin, Cotonou, Benin; 3 Laboratory of Biology and Molecular Typing in Microbiology (LBTMM), Faculty of Sciences and Technology (FAST), University of Abomey - Calavi, Cotonou, 05 BP 1604 Benin; 4 Molecular Biology Laboratory, Muraz Center, Higher Institute of Health Sciences (INSSA), Nazi Boni University, Bobo-Dioulasso, Burkina Faso; 5 Health Program Fighting Against AIDS in Benin (PSLS), Health Ministry of Benin, Cotonou, Benin; 6 Laboratory of Emerging and Re-emerging Pathogens (LaPathER), Doctoral School of Health Sciences, Nazi BONI University, Bobo-Dioulasso, BP: 2161, Burkina Faso

**Keywords:** Benin – PMTCT – HIV, Early diagnosis, Transmission rate

## Abstract

**Background:**

The implementation of the WHO’s 2015 recommendations in Benin, requires an assessment of the progress made over time in preventing the transmission of the infection to exposed-infants, and the identification of its determinants.

**Methods:**

This was a retrospective study of HIV-1 exposed-infants who underwent PCR between the 6^th^ and 8^th^ weeks of life. Early diagnostic tests were performed using the Abbott *m*2000 RealTime platform. Comparison of proportions tests (analysis of the significance of the difference in prevalence) with an error threshold of 5% were used to assess the determinants of the transmission. Statistical analysis was performed using R statistical software, version 4.1.3.0.

**Results:**

A total of 5,312 infants benefited from early diagnosis by PCR between 2016 and 2021. Among them, 52% are males, tritherapy before pregnancy was the majority treatment used by mothers (30.6%) and monotherapy that of newborns (70%). Mixed breastfeeding is the feeding method with the highest prevalence. The overall transmission rate was 3.4% over the six years. The highest prevalence was achieved in 2018 (4.2%) and the lowest in 2021 (2.7%). The prevalence was lower when mothers were on tritherapy before pregnancy. The determinants of transmission were: mixed breastfeeding, lack of treatment in mothers (22.4%), lack of treatment in infants (19.7%), undefined treatments or absence of treatment in the mother-child pair.

**Conclusion:**

This study shows the contribution over time of the PMTCT program to reducing HIV transmission among exposed-infants and also underlines the need for proper conduct of treatment in any women of childbearing age.

## Introduction

Around 130,000 children have been newly infected with Human Immunodeficiency Virus (HIV) worldwide, and sub-Saharan Africa, in particular, accounts for more than 2/3 of them [1]. Around 90% of infections in children are caused by vertical transmission [2]. Unless action is taken to reduce transmission in this region of Africa, 30–40% of infants born to mothers living with HIV will become infected [2]. This shows the importance of these programs in reducing the incidence of infection in children. Regional disparities are noted in the coverage of prevention of mother-to-child transmission (PMTCT) on the continent. In East and Southern Africa, for example, coverage is 89%, compared with 60% in West and Central Africa, where efforts still need to be made to achieve the objectives set by the joint United Nations Program on HIV/AIDS (UNAIDS) for 2025 [1]. The objective of PMTCT is to reduce transmission to less than 5% in order to impact on 90% of new childhood infections. According to 2021 estimates from UNAIDS, the estimated rate of PMCT in sub-Saharan Africa is 10.52% with 21.39% in the West and Central regions [3].

In Benin, the PMTCT program began in 2004 and has intensified over time in order to reduce the prevalence of this infection in children. Indeed, pregnant women who screen positive for HIV infection are put on treatment and then enrolled in program to prevent mother-to-child transmission. Antiretroviral therapy (ART) for these women consists of either tritherapy before pregnancy (for those screened before pregnancy), during pregnancy or childbirth for those tested positive after becoming pregnant. Antiretroviral therapy is instituted for life. This treatment consists of two nucleoside reverse transcriptase inhibitors and a non-nucleoside reverse transcriptase inhibitor or an integrase inhibitor (TDF/ABC + 3TC + EFV or TDF/ABC + 3TC + DTG + folic acid). Before giving birth, women are advised on the choice of breastfeeding method (exclusive maternal, protected maternal, or mixed) as well as on newborn prophylaxis. The current prophylaxis regimens in Benin consist of monoprophylaxis (zidovudine syrup or nevirapine syrup) when the risk of transmission is low or biprophylaxis (zidovudine + nevirapine) in case of high risk of transmission [4–6]. Later, between the sixth and eighth weeks of life, infants are tested for an early diagnosis of HIV infection. According to the S2-2021 monitoring report, approximately 2,392 children are living with HIV in Benin [7]. The national prevalence of perinatal transmission was 2.1% in S2-2021. However, according to UNAIDS estimates for 2022, the vertical transmission prevalence for all factors combined is 10.8% [8]. Of course, progress remains to be made to achieve the objectives, but it is possible by drawing inspiration from the countries for which the World Health Organization (WHO) has validated the elimination of transmission [9, 10]. These examples clearly demonstrate that the combination of both financial and material support from partners with a well-developed strategy, adequate monitoring of indicators, individual efforts, and good support from political leaders can all help to achieve these objectives.

In Benin, there is very little data in the literature on the progress made in reducing perinatal transmission of this infection, although various reports are available and can be consulted at the level of the Health Program to Fight Acquired Immuno Deficiency Syndrome (AIDS). This study aims to present an overview of the progress made in the vertical transmission of HIV infection, six years after the implementation of the 2015 WHO recommendations, to deduce the determinants and successful combinations.

## Methods

### Study population

This was a retrospective study carried out at the National Reference Laboratory (NRL) for HIV located in the Littoral department. The sampling method was an exhaustive and systematic selection of all infants aged ≤ 8 weeks, born to HIV-positive mothers who gave birth under PMTCT protocol between January 1, 2016 and December 31, 2021 in the Atlantic and Littoral departments.

All participants who did not meet this age criterion or without data were not included.

### Sampling and data collection

Systematic and exhaustive sampling of all infants meeting inclusion criteria was used. Data were extracted from the database of the National Program for the Prevention of Mother-to-Child Transmission. The main dependent variable was the HIV status of the infants (positive or negative), and the independent variables were age (weekdays), sex, feeding mode, ART protocol applied to mothers (tritherapy before pregnancy, tritherapy during pregnancy, tritherapy during childbirth, no treatment, treatment not specified), the PMTCT protocol applied to infants (monoprophylaxis, biprophylaxis, none, not specified).

### Biological analysis

A blood sample was taken from infants by prick of the finger or heel. Five drops of blood were collected on blotting paper or Dried Blood Spot (DBS) card (Wattman 903). These DBS cards were dried for at least 3 h at room temperature, on the drying device, packaged with a desiccant, then sent to the National Reference Laboratory (NRL) for HIV-1 proviral DNA detection by polymerase chain reaction (PCR). When HIV-1 proviral DNA was detected in a sample, the infants were declared HIV-1 positive, and negative otherwise.

DNA detection was performed with Abbott *m*2000 RealTime HIV-1 assay (Abbott Molecular) for early diagnosis of HIV-1 infection in infants. Extraction was performed according to the manufacturer’s recommendations using the Abbott *msample* preparation kit. After extraction, amplification was carried out with the Abbott *m*2000rt thermocycler. The detection limit of this method was 40 copies/ml (1.6 log _10_ /ml).

### Statistical analyzes

The data were collected in an Excel ^®^^2016^ database. Statistical analyses were carried out using the R statistical software, version 4.1.3.0. A descriptive analysis of the data distribution thus constituted across the different variables made it possible to understand better the population studied. Univariate analysis was used to determine the association between PMTCT interventions and HIV transmission. Multivariate tests for comparison of proportions (analysis of the significance of the difference in prevalence) with an error threshold of 5% were used to evaluate the effectiveness of treatments and diet on the transmission of HIV infection from mother to child. A difference was found to be statistically significant when the value of “z” is greater than “t” (1.96 reduced centered normal law).

### Ethical considerations

The study protocol was approved by the AIDS Health Program in Benin. A letter of authorization for data collection was obtained from the program authorities and the National Reference Laboratory. The protocol was also approved by the National Ethics Committee for Health Research (CNERS) under the trial number 30 of August 30, 2020 (Reference N°: 111/MS/DRFMT/CNERS/SA). All information regarding the study subjects was confidential.

## Results

### Main features

This study included 5,312 infants born to HIV-positive mothers. More than half (52.2%) of the infants sampling were males (Table 1).

**Table 1:** Patient’s distribution by sex

Sex	*N*	Distribution (%)
F	2,539	47.8%
M	2,773	52.2%
Total	5,312	100.0%

### Prevalence of transmission over time

Overall, the number of PCR tests performed has increased over the years, from 821 (2016) to 1,042 (2019). We found a combined prevalence of 3.4% over the six years. The prevalence of HIV infection among children has declined over time. The highest prevalence was recorded in 2018 (4.2%) and the lowest in 2021 (2.7%). (Table 2)

**Table 2:** HIV prevalence by year

	Distribution			
Year	Negative	Positif	Total	% positive
2016	794	27	821	3.3
2017	940	32	972	3.3
2018	952	42	994	4.2
2019	1,004	38	1,042	3.6
2020	834	24	858	2.8
2021	608	17	625	2.7
Total	5,132	180	5,312	3.4

### Prevalence of HIV transmission by diet mode

The breastfeeding method mostly chosen by mothers is protected breastfeeding (66.3%). Mixed breastfeeding has a higher prevalence of infection (13.7%) than other methods. However, its population is not large (2.3% of all infants) (Table 3).

**Table 3:** Prevalence of HIV transmission by diet

Diet	Prevalence of transmission according to diet			
	Negative	Positive	Total (%)	% positive
Substitute foods	318	11	329 (6.19)	3.3
Breastfeeding	988	52	1,040 (19.57)	5.0
Protected breastfeeding	3,448	74	3,522 (66.30)	2.1
Mixed breastfeeding	107	17	124 (2.33)	13.7
Others	140	13	153 (2.88)	8.5
Not specified	131	13	144 (2.7)	9.0
Total	5,132	180	5,312	3.4

### Prevalence of transmission according to mother’s treatment

The predominant treatment protocol among mothers is tritherapy before pregnancy (30.6%). There is a high proportion of infants born without a treatment protocol (22.4%). The prevalence is lower (1.9%) when mothers received tritherapy before pregnancy, compared to when they received tritherapy during pregnancy (2.3%) (Table 4).

**Table 4:** Prevalence by mother’s treatment

	Prevalence of transmission according to mother’s treatment				
Mother’s treatment	Negative	Positive	Total (%)	% positive	% global
None	191	55	246 (4.63)	22.4	3.4
Others	488	19	507 (9.54)	3.7	3.4
Not specified	378	18	396 (7.45)	4.5	3.4
Triprophylaxis	1,022	23	1,045 (19.67)	2.2	3.4
Triple therapy during pregnancy	1,459	34	1,493 (28.11)	2.3	3.4
Triple therapy before pregnancy	1,594	31	1,625 (30.6)	1.9	3.4
Total	5,132	180	5,312	3.4	

However, we noticed that the difference is not statistically significant between mother’s who received therapy before pregnancy and those who received it during pregnancy (z = 0.72). We also found statistical differences between tritherapy before pregnancy and when treatment was not specified or none treatment respectively (z = 2.40 and z = 7.64) (Table 5).

**Table 5:** Comparison of proportion of maternal treatment

	|P1 - P2|	A = P1(1-P1)/n1	B = P2(1 P2)/n2	A + B	$\:\surd\:$A + B)	Z=|P1 - P2|/ $\:\surd\:$(A + B)	T bilateral reduced centered normal distribution 95%
Tritherapy before pregnancy vs. tritherapy during pregnancy	0.0037	0.0000	0.0000	0.0000	0.0051	0.72	1.96
Tritherapy before pregnancy vs. triprophylaxis	0.0029	0.0000	0.0000	0.0000	0.0057	0.52	1.96
Tritherapy before pregnancy vs. not specified	0.0264	0.0000	0.0001	0.0001	0.0110	2.40	1.96
Tritherapy before pregnancy vs. others	0.0184	0.0000	0.0001	0.0001	0.0091	2.02	1.96
Tritherapy before pregnancy vs. none	0.2045	0.0000	0.0007	0.0007	0.0268	7.64	1.96
Triple therapy during pregnancy vs. triprophylaxis	0.0008	0.0000	0.0000	0.0000	0.0060	0.13	1.96
Triple therapy during pregnancy vs. not specified	0.0227	0.0000	0.0001	0.0001	0.0112	2.03	1.96
Triple therapy during pregnancy vs. others	0.0147	0.0000	0.0001	0.0001	0.0093	1.58	1.96
Triple therapy during pregnancy vs. none	0.2008	0.0000	0.0007	0.0007	0.0268	7.48	1.96
Triprophylaxis vs. not specified	0.0234	0.0000	0.0001	0.0001	0.0114	2.05	1.96
Triprophylaxis vs. others	0.0155	0.0000	0.0001	0.0001	0.0096	1.61	1.96
Triprophylaxis vs. none	0.2016	0.0000	0.0007	0.0007	0.0269	7.48	1.96

### Prevalence of transmission according to exposed infant’s prophylaxis mode

Monoprophylaxis was the best protocol chosen by parents for prophylaxis (70.1%). When the infant is on monoprophylaxis, the prevalence is 2.5%, and without treatment, it is 19.4% (Table 6).

**Table 6:** Prevalence of transmission according to infants exposed prophylaxis mode

	Prevalence of transmission according to childhood prophylaxis				
Infants prophylaxis mode	Negative	Positive	Total (%)	% positive	% global
None	50	12	62 (1.16)	19.4	3.4
Others	563	32	595 (11.20)	5.4	3.4
Biprophylaxis	368	12	380 (7.15)	3.2	3.4
Monoprophylaxis	3,634	93	3,727 (70.16)	2.5	3.4
Not specified	517	31	548 (10.31)	5.7	3.4
Total	5,132	180	5,312	3.4	

Comparison on prophylaxis therapy showed a statistically significant difference between monoprophylaxis and no treatment or not defined treatment (respectively z = 3.36 and z = 3.10) (Table 7).

**Table 7:** Comparison of infants’ prophylaxis treatment

	|P1 - P2|	A= P1(1-P1)/n1	B= P2(1-P2)/n2	A + B	$\:\surd\:$(A + B)	Z= |P1 -P2| /$\:\surd\:$(A + B)	T bilateral reduced centered normal distribution 95%
Not specified vs. none	0.1370	0.0001	0.0025	0.0026	0.0511	2.68	1.96
Not specified vs. other	0.0028	0.0001	0.0001	0.0002	0.0135	0.21	1.96
Not specified vs. biprophylaxis	0.0250	0.0001	0.0001	0.0002	0.0133	1.87	1.96
Not specified vs. monoprophylaxis	0.0316	0.0001	0.0000	0.0001	0.0102	3.10	1.96
Monoprophylaxis vs. none	0.1686	0.0000	0.0025	0.0025	0.0502	3.36	1.96
Monoprophylaxis vs. other	0.0288	0.0000	0.0001	0.0001	0.0096	3.00	1.96
Monoprophylaxis vs. biprophylaxis	0.0066	0.0000	0.0001	0.0001	0.0093	0.71	1.96
Biprophylaxis vs. none	0.1620	0.0001	0.0025	0.0026	0.0510	3.18	1.96
Biprophylaxis vs. other	0.0222	0.0001	0.0001	0.0002	0.0129	1.72	1.96

### Prevalence of transmission according to mother-child pair therapy

Monoprophylaxis in infants combined with tritherapy before pregnancy in the mother is the best option to reduce the proportion of HIV infection (1.4%) (Table 8).

**Table 8:** Prevalence of transmission by mother-child pair treatment

		Prevalence of transmission by mother’s treatment / infants treatment			
Mother’s PMTCT	Infants	Negative	Positive	Total	% positive
None	None	5	5	10	50.0
	Others	29	11	40	27.5
	Biprophylaxis	24	3	27	11.1
	Monoprophylaxis	112	26	138	18.8
	Not specified	21	10	31	32.3
		488	19	507	3.7
Others	None	10	3	13	23.1
	Others	91	8	99	8.1
	Biprophylaxis	72	0	72	0.0
	Monoprophylaxis	270	7	277	2.5
	Not specified	45	1	46	2.2
		378	18	396	4.5
Not specified	None	3	0	3	0.0
	Others	14	0	14	0.0
	Biprophylaxis	8	3	11	27.3
	Monoprophylaxis	182	7	189	3.7
	Not specified	171	8	179	4.5
		1,022	23	1,045	2.2
Triple prophylaxis	None	12	1	13	7.7
	Others	88	2	90	2.2
	Biprophylaxis	87	2	89	2.2
	Monoprophylaxis	748	12	760	1.6
	Not specified	87	6	93	6.5
		1,459	34	1,493	2.3
Triple therapy during pregnancy	None	9	0	9	0.0
	Others	165	5	170	2.9
	Biprophylaxis	100	2	102	2.0
	Monoprophylaxis	1,084	24	1,108	2.2
	Not specified	101	3	104	2.9
		1,594	28	1,622	1.7
Triple therapy before pregnancy	None	11	3	14	21.4
	Others	176	6	182	3.3
	Biprophylaxis	77	2	79	2.5
	Monoprophylaxis	1,238	17	1,255	1.4
	Not specified	92	0	92	0.0

## Discussion

Our observations focused on 5,312 infants born to HIV- infected mothers, spread over six years with a decrease in the number of infants between 2020 and 2021. This reduction is justified by the improvement of the technical platform of the sites through the purchase and implementation Point of Care equipment (GeneXpert) is available. Thus, PCR tests have been decentralized at the national level, which has improved health coverage, the time taken to provide results and the treatment of infants.

The feeding method preferentially chosen by the participants’ mothers has protected breastfeeding (66.3%). This same observation was made in a previous study in Benin [11]. In Africa where most families live in communities, it is difficult to explain why a child would not be breastfed when the mother is apparently in good health and free of any HIV infection [12]. Mixed breastfeeding is the one for which the vertical transmission prevalence was the highest (13.7%). This result is consistent with those obtained by different studies. A study conducted in North-West Ethiopia showed that there is a statistically powerful association between mixed breastfeeding and transmission of infection to infants [13]. In China, the prevalence of transmission of infection through mixed breastfeeding among infants was 60.00% [14]. This high proportion of transmission may be explained by the more frequent occurrence of conditions such as subclinical mastitis or breast abscesses under this type of breastfeeding. To date, there is no scientific certainty to explain this situation [15, 16].

The PMTCT protocol mainly selected by mothers is tritherapy before pregnancy in 30.6% of cases. The positivity proportion was 2.2%, 22.4% and 4.7% respectively among mothers on triprophylaxis, without treatment and those in whom treatment is not defined. In Bing Li’s study, the proportion were 9.23% and 20.93% under treatment and without treatment, respectively [14]. Among mothers, triple therapy before pregnancy has a lower prevalence (1.9%) than tritherapy during pregnancy (2.3%), although this difference was not statistically significant (z = 0.72). A Zambian study demonstrated that the duration of treatment before delivery plays a key role in MTCT [17]. However, we found a statistically significant difference between tritherapy before pregnancy and no treatment or no defined treatment (z = 7.64 and z = 2.40, respectively). The same applies to the tritherapy during pregnancy and the absence of treatment or when the treatment is not defined (respectively z = 7.48 and z = 2.03). In Belay B’s study, the absence of PMTCT in mothers was associated with a higher risk of transmission [18].

Monoprophylaxis was chosen by 70% of mothers as prophylactic treatment for their infants. This protocol was certainly the best treatment for this population with a 2.5% risk of infection. Whereas in Yu Dong’s study the administration of zidovudine alone was associated with a higher risk of MTCT [19 ]. Among infants, we noted that those who did not receive prophylaxis were more likely to have an infection (19.4%) than others. This proportion is lower than those obtained in other studies, which can be justified because only 1% of participants are affected [19, 20]. We also found a statistically significant difference between monoprophylaxis and no treatment as well as when treatment is not defined (respectively z = 3.36 and z = 3.10). In Lubumbashi, Democratic Republic of Congo, a study found that infants who received Nevirapine had a lower risk of MTCT (7%) than those who did not 66% [20].

Early detection of HIV infection, over the past six years shows a pooled transmission prevalence of 3.4% with a minimum of 2.7% in 2021 and a maximum of 4.2% in 2018. As reported in North-East Nigeria (from 14.3% in 2008 to 4.9% in 2014), Senegal (14.8–4.1% between 2008 and 2015), Burkina Faso (10.4% in 2006 to 0% in 2015), Ethiopia (2.9–0.9% between 2016 and 2020) a decrease in the proportion of MTCT over time has been observed, linked to improvements of the protocols put in place [21–24]. The overall prevalence observed in our study is lower than that reported elsewhere: Ethiopia (5.9% and 15.7%, Cameroon (5%), Benin (6.59%), Burkina Faso (3.6%), Nigeria (4.8%) Tchad (14.8% ) [25–31]. We believe that this performance was made possible thanks to the strong involvement of the Claudine TALON Foundation in the fight against MTCT [32].

The results obtained in our study suggest that infants who have not received treatment and whose mothers have not received it either are 50% more likely to be infected with HIV. Other studies have obtained higher rates [28, 33]. When tritherapy before pregnancy and monoprophylaxis were combined, the prevalence of infection was lower than when tritherapy during pregnancy was combined with monoprophylaxis (1.4% vs. 2.2%). Regardless of the treatment received by the mothers, infants on biprophylaxis have a positivity rate equal to or greater than 2%. Also at the scale of these participants, HIV infection was more dependent on the therapeutic protocols of the mothers than on those of the infants. Adequate and continuous therapeutic care for the mother-child pair improves the prevalence of infection.

The determinants of MTCT deduced from our data analysis are similar to those observed in the literature: mixed breastfeeding, absence of treatment or undefined treatment for both mothers and infants because these factors increase the risk of transmission of infection in infants born to HIV-positive mothers [18, 34].

## Conclusion

Data from this study showed that the prevalence of HIV transmission from mothers living with HIV to their infants has decreased over the years and through interventions. A residual rate of MTCT remains in these two departments despite improvements over the last six years. Everyone’s commitment is essential to achieving UNAIDS’ 2025 targets.

### Strengths and limitations

Our results show the progress made by the government and stakeholders at different levels to achieve the elimination of mother-to-child transmission while providing a better understanding of the determinants. In addition to the large size of our sample, which provides a good representation of the population studied, this is a resource that could guide policy-makers and funding agencies in development of relevant strategies for the elimination of mother-to-child transmission.

The main limitation is that the information was collected from PCR request forms and depends on the prescribers’ correct completion of these forms.

## Data Availability

All data generated and analysed during this study are not publicly available. Study data is only available to scientific collaborators. Data are available upon on request from the corresponding author, Edwige Hermione DAGBA GBESSIN. Data may also be available upon request. All data sharing is subject to the National Ethics Committee for Health Research.

## Abbreviations

HIV: Human immunodeficiency virus
PMTCT: Prevention of mother-to-child transmission
WHO: World Health Organisation
PCR: Polymerase chain reaction
UNAIDS: United Nations Programme on HIV/AIDS
EFV: Efavirenz
TDF: Tenofovir
ABC: Abacavir
3TC: lamivudine
DTG: Dolutegravir
AIDS: Acquired immune deficiency syndrome
NRL: National reference laboratory
DBS: Dried blood spot
ART: antiretroviral treatment
DNA: Deoxyribonucleic acid
CNERS: Ethics committee for health research
MTCT: Mother to child transmission
